# Genetically Depauperate in the Continent but Rich in Oceanic Islands: *Cistus monspeliensis* (Cistaceae) in the Canary Islands

**DOI:** 10.1371/journal.pone.0017172

**Published:** 2011-02-14

**Authors:** Mario Fernández-Mazuecos, Pablo Vargas

**Affiliations:** Real Jardín Botánico, CSIC, Madrid, Spain; Prognosys Biosciences, United States of America

## Abstract

**Background:**

Population genetic theory holds that oceanic island populations are expected to have lower levels of genetic variation than their mainland counterparts, due to founder effect after island colonization from the continent. *Cistus monspeliensis* (Cistaceae) is distributed in both the Canary Islands and the Mediterranean region. Numerous phylogenetic results obtained in the last years allow performing further phylogeographic analyses in *Cistus*.

**Methodology/Principal Findings:**

We analyzed sequences from multiple plastid DNA regions in 47 populations of *Cistus monspeliensis* from the Canary Islands (21 populations) and the Mediterranean basin (26 populations). The time-calibrated phylogeny and phylogeographic analyses yielded the following results: (1) a single, ancestral haplotype is distributed across the Mediterranean, whereas 10 haplotypes in the Canary Islands; (2) four haplotype lineages are present in the Canarian Islands; (3) multiple colonization events across the archipelago are inferred; (4) the earliest split of intraspecific lineages occurred in the Early to Middle Pleistocene (<930,000 years BP).

**Conclusions/Significance:**

The contrasting pattern of cpDNA variation is best explained by genetic bottlenecks in the Mediterranean during Quaternary glaciations, while the Canarian archipelago acted as a refugium of high levels of genetic diversity. Active colonization across the Canarian islands is supported not only by the distribution of *C. monspeliensis* in five of the seven islands, but also by our phylogeographic reconstruction in which unrelated haplotypes are present on the same island. Widespread distribution of thermophilous habitats on every island, as those found throughout the Mediterranean, has likely been responsible for the successful colonization of *C. monspeliensis*, despite the absence of a long-distance dispersal mechanism. This is the first example of a plant species with higher genetic variation among oceanic island populations than among those of the continent.

## Introduction

Islands constitute a focus of research interest in plant evolutionary biology, given their limited area and varying degrees of isolation from nearby continents. Continental islands are located on continental shelves, and were isolated from the continent by means of rising sea level or/and by tectonic processes. These islands may have been recurrently connected to the continent by land bridges due to fluctuating sea levels. On the contrary, oceanic islands arise from the ocean floor, are usually of volcanic origin and have virtually no terrestrial life in origin [1], [2]. Because they furnish clear-cut spatial and temporal limits, oceanic islands are considered to be living laboratories for evolution. That is why oceanic islands provide ideal systems to investigate historical colonization and evolutionary patterns in plants [3]. Speciation processes giving rise to endemic species and lineages on oceanic islands have been widely discussed, leading to alternative models of evolution [2], [4], [5], [6], [7], [8]. Less attention has been paid to populations of species distributed both on continents and oceanic islands. Nonetheless, populations of the same species distributed in insular and mainland areas can provide key insights into microevolutionary processes underlying recent colonization and early stages of differentiation. As a general pattern, lower levels of genetic variation are expected in island populations as compared to mainland populations due to founder effects and restricted gene flow [9]. This depauperation may bring about an increased propensity for extinction and compromised evolutionary potential in island populations. Early studies of genetic variation in mainland and island populations included only one study of oceanic islands [9], [10]. More recent examples of island-mainland comparisons have been reported, most of which also focused on continental islands [11], [12], [13], [ but see 14], [15]. These examples generally agree with the expectation of higher genetic variation in mainland populations. The finding of higher variation in continental islands has been attributed to multiple continent-to-island introductions, genetic bottlenecks in the continent, or island-to-continent colonization [13], [16], [17]. To our knowledge, higher genetic variation has not been reported for oceanic islands, where particularly strong genetic bottlenecks are expected due to isolation and the prevalence of single introduction events [18].

The genus *Cistus* L. (Cistaceae) comprises 21 species of primarily Mediterranean distribution. The highest species diversity is found in the western Mediterranean, which has 14 species. Seven species are present in the Canary Islands (Macaronesian region). Five of them are endemic and form a sublineage within the purple-flowered clade [19], [20], while the white-flowered *C. monspeliensis* and *C. ladanifer* are also widely distributed Mediterranean elements. Although a close relationship between the Mediterranean and Macaronesian floras is well established [8], [21], [22], [23], there are few reports of phylogeographic and population genetic analysis of species that are present in both floristic regions [but see 14], [24], [25] , such as these two *Cistus* species. This might be due to the risk of including plant populations introduced since the first human (*guanches*) colonization, coupled with more interest in the endemic element [4], [6], [26].

In particular, it is not clear whether the occurrence of *C. monspeliensis* in the Canary Islands is the result of natural or human-mediated introduction [19]. Some white-flowered species of *Cistus* with a local presence in Macaronesia are considered introduced species, such as *C. ladanifer* in the Canary Islands [27] and *C. psilosepalus* and *C. salviifolius* in Madeira [28]. In contrast, it has been suggested that *C. monspeliensis* is a native species in the Canary Islands [23], although no morphological differentiation has been reported for most of the populations that supports this hypothesis (but see *C. grancanariae* [29]). A recent phylogeographic analysis found no nucleotide variation in 26 Mediterranean populations of *C. monspeliensis* after screening 17 cpDNA regions, which indicated that there was a rapid dispersal across the Mediterranean after species formation in the Pleistocene [30]. The question remains as to whether Canarian populations share the same Mediterranean genotype or show some degree of genetic exclusiveness, the latter of which would support the native status hypothesis.

The plastid genome is structurally stable, haploid, non-recombinant and maternally inherited in *Cistus* [31]. Accordingly, it has been used to infer phylogeographic and seed colonization patterns for this genus [20], [30], [31]. In the present study, we analyzed cpDNA haplotypes of insular and continental populations of *C. monspeliensis*. Our goal was three-fold: (1) to compare levels of genetic variation; (2) to determine native versus human-introduced status for the Canarian populations; and (3) to reconstruct the colonization history in the Canary Islands.

## Methods

### Study species

*Cistus monspeliensis* L. is a lowland shrub displaying a rather continuous distribution in the Mediterranean basin. It usually occurs on poor soils of the thermomediterranean vegetation belt (600–800 m) on both calcareous and acidic substrates. In the Canary Islands, it occurs in the understory and successional scrub of thermophilous forests, laurel forests and *Pinus canariensis* woodlands of five islands: Tenerife, Gran Canaria, La Palma, El Hierro and La Gomera [23]. It has also been reported in Madeira, but it is probably no longer present in this archipelago [28].

### Sample strategy and DNA sequencing

A total of 47 populations of *Cistus monspeliensis* were sampled to cover its distributional area: 21 populations from the Canary Islands (53 individuals; Table 1), plus 26 Mediterranean populations (26 individuals) previously analyzed [30] (Table S1). All new individuals were collected in the field and dried in silica gel. First, a pilot study was performed to find the most variable sequences among 17 plastid DNA regions previously used in phylogenetic and phylogeographic analyses [see 30 for details].

**Table 1:** Canarian *Cistus monspeliensis* populations used for plastid sequencing of the *trn*S-*trn*G and *psb*K-*trn*S regions, and haplotypes found in sequenced individuals.

Population number	Locality (number of individuals)	Voucher	Haplotype codes
1	El Hierro, La Peña (3)	C. García-Verdugo 39CG05 (MA)	B/B/B
2	El Hierro, Valverde (3)	C. García-Verdugo 41CG05 (MA)	F/J/J
3	La Palma, Barranco Garome (3)	C. García-Verdugo 34CG05 (MA)	C/C/D
4	La Palma, La Tosca (3)	B. Guzmán 142BGA04 (MA)	C/C/C
5	La Palma, Los Llanos – Santa Cruz (3)	V. Valcárcel 63VV04 (MA)	C/C/D
6	La Palma, Villa de Mazo (1)	P. Vargas 254PV02 (MA)	C
7	La Palma, Barranco Seco (3)	C. García-Verdugo 26CG05 (MA)	D/D/D
8	La Gomera, Arure (3)	C. García-Verdugo 19CG05 (MA)	H/K/K
9	La Gomera, Alto de Garajonay (3)	A. Herrero AH2443 (MA)	J/J/J
10	La Gomera, Jaragán (2)	C. García-Verdugo 10CG05 (MA)	J/J
11	Tenerife, Barranco de las Ánimas (1)	B. Guzmán 12BGA05 (MA)	B
12	Tenerife, Villa de Arico (3)	P. Vargas 42PV05 (MA)	E/E/H
13	Tenerife, road to Teide (1)	B. Guzmán 14BGA05 (MA)	G
14	Tenerife, Aguamansa (1)	C. García-Verdugo 8CG05 (MA)	J
15	Tenerife, Güímar (3)	C. García-Verdugo 54PV05 (MA)	J/J/J
16	Tenerife, Igüeste de San Andrés (3)	P. Vargas 56PV05 (MA)	B/B/B
17	Gran Canaria, Embalse del Mulato (2)	B. Guzmán 1BGA05 (MA)	G/G
18	Gran Canaria, Artenara (3)	P. Vargas 61PV05 (MA)	B/G/J
19	Gran Canaria, road GC-605 (3)	B. Guzmán 3BGA05 (MA)	I/I/J
20	Gran Canaria, San Bartolomé de Tirajana (3)	B. Guzmán 6BGA05 (MA)	G/G/G
21	Gran Canaria, Roque Nublo (3)	P. Vargas 169PV08 (MA)	G/G/J

Procedures used for amplification and sequencing of DNA regions followed Fernández-Mazuecos and Vargas [30]. After identifying two DNA regions with high sequence variation (*trn*S-*trn*G and *psb*K-*trn*S), we extended the sequencing to every population (1–3 individuals per population in the Canary Islands). The same DNA regions were also sequenced from the other eleven species of the white-flowered lineage [19], [32] and two purple-flowered species as the outgroup (Table S1), to reconstruct phylogenetic relationships of the cpDNA haplotypes (see below). A recently described species (*C. grancanariae* [29]) related to *C. monspeliensis* needs further taxonomic validation. Nevertheless, a preliminary study rendered no sequence variation between the two taxa for the two cpDNA regions (Fernández-Mazuecos & Vargas, unpublished). All new sequences have been deposited in GenBank (see Table S1 for accession numbers).

### Haplotype data analyses

All *trn*S-*trn*G and *psb*K-*trn*S sequences were assembled in Geneious Pro 4.8.3 [33] and aligned using ClustalW 2.0.12 [34]. Further adjustments were made by visual inspection. Species most closely related to *C. monspeliensis* according to previous results were included as the outgroup. Genealogical relationships among haplotypes based on nucleotide substitutions were inferred using the statistical parsimony algorithm [35], as implemented in TCS 1.21 [36]. The maximum number of differences resulting from single substitutions among haplotypes was calculated with 95% confidence limits, treating gaps as missing data.

Phylogenetic relationships were also assessed using maximum parsimony (MP) and Bayesian inference (BI). Analyses were conducted combining sequences of the two DNA regions representing the haplotypes of *C. monspeliensis* plus the other eleven white-flowered and two purple-flowered *Cistus* species. MP analyses were performed using PAUP* 4.0b10 [37], with the following parameters for the heuristic search: 1000 random addition replicates holding 100 trees per replicate, tree-bisection-reconnection (TBR) branch swapping and the options Multrees and Steepest Descent in effect. Robustness of clades was estimated using 1,000,000 bootstrap replicates (fast stepwise-addition [38]). BI was implemented in MrBayes v3.1.2 using two identical searches with 10 million generations each (chain temperature  = 0.2; sample frequency  = 100). The simplest model of sequence evolution that best fits the sequence data (GTR+G) was determined under the Akaike Information Criterion (AIC) in jModeltest 0.1.1 [39], [40]. Probabilities converged on the same stable value after c. 20,000 generations in both runs. A 50% majority rule consensus tree was calculated to obtain the Bayesian estimate of phylogeny [see 19 for details].

### Genetic diversity

For each island, we calculated the number of haplotypes based on nucleotide substitutions (*h*), number of private haplotypes (*ph*) and haplotypic diversity (*H*) [41]. The same parameters were computed for the Mediterranean basin and the Canary Islands as a whole. Using previously published cpDNA data [20], [30], [31], h and H were calculated for other monophyletic *Cistus* species and lineages recently differentiated in the Mediterranean and the Canary Islands. Given that the *trnS-trnG* spacer has been employed in all published analyses of *Cistus*, *h* and *H* were also calculated for this region separate. In all cases, calculations were performed in DnaSP v5 [42]. In this software, sites with alignment gaps (or missing data) are excluded from calculations. Therefore, we eliminated certain samples (one individual of *C. creticus* and one individual of *C. symphytifolius* [20]) with indels or missing data in variable sites in order to properly infer substitution-based diversity parameters.

### Among-island genetic differentiation

The nearest-neighbour statistic (S_nn_) was calculated to assess genetic differentiation in *C. monspeliensis* due to isolation in different islands. This statistic is a measure of how often the “nearest neighbours” (in sequence space) of sequences are from the same locality in the geographic space [43]. S_nn_ is expected to approach one when two partitions (areas, localities) of a dataset form highly differentiated populations, and one-half when they are part of a single panmictic population. The dataset was divided into five partitions corresponding to the five islands with presence of the species. S_nn_ was calculated using DnaSP v5, and permutation tests with 1000 replicates were performed to evaluate significance of the obtained values.

### Bayesian dating and phylogeographic reconstruction

In order to infer divergence times among *C. monspeliensis* lineages and reconstruct its colonization history across the Canarian archipelago, the dataset was analyzed using a relaxed Bayesian approach as implemented in BEAST v.1.6.1 [44], [45]. We employed the spatial diffusion methodogy, a recently developed approach to phylogeography aimed to identify the ancestral geographical history of a sample of molecular sequences [46], [47], [48]. Unlike other modern approaches to phylogeographic inference [49], these models do not infer the demographic history of populations, but they are able to reconstruct historical movements of or between populations [46]. Therefore, this methodology is appropriate for our inference of colonization history across an oceanic archipelago. We employed *C. populifolius* as outgroup based on previous phylogenies [30], [32], and the HKY substitution model was chosen following jModeltest result. We implemented a relaxed molecular clock, with an uncorrelated lognormal distribution for the substitution rate variation, and a coalescent model with constant size was assumed as tree prior. The root height was modelled as a normal distribution with mean  = 1.13 Ma, based on the divergence time between *C. monspeliensis* and *C. populifolius* previously estimated using fossil calibrations [30]. The uncertainty on this calibration point (95% highest posterior density interval 0.24–2.41 Ma [30]) suggests a standard deviation  = 0.66. However, a stronger prior is desirable for the root height in order to estimate evolutionary rates, and thus, with values above 0.20, the MCMC failed to converge to a coherent result, with all node ages approaching zero. Therefore, we chose 0.20 as an appropriate value of standard deviation, but we acknowledge that the uncertainty on divergence times may be higher than shown by our results. In any case, our ultimate goal was to find a reliable upper bound rather than a precise dating. The colonization history was reconstructed using a Bayesian phylogeographic framework [47]. We defined six areas (the five islands plus the Mediterranean region), and they were mapped with a discrete phylogeographic analysis using a standard continuous-time Markov chain (CTMC). As suggested by [47], we also implemented a Bayesian stochastic search variable selection (BSSVS) procedure to identify parsimonious descriptions of the diffusion (colonization) process. We employed all the standard parameters suggested in the authors' web site (http://beast.bio.ed.ac.uk/Tutorials). Two MCMC analyses were run for 10 million generations, sampling every 1000th generation. Analysis with Tracer 1.4 [50] confirmed convergence among chains and adequate sample size. Both chains were combined using LogCombiner 1.4.8 after discarding the first 10% of sampled generations as burn-in, and trees were summarized in a maximum clade credibility (MCC) tree obtained in TreeAnotator 1.6.1 and visualized in FigTree 1.3.1. Finally, a Bayes factor (BF) test was performed to identify rates (colonization routes) that are frequenly invoked to explain the diffusion process. Rates yielding a BF>3 were considered as well supported, and were converted into a KML file suitable for visualization in Google Earth [47].

## Results

### Haplotype analysis

**Figure 1:**
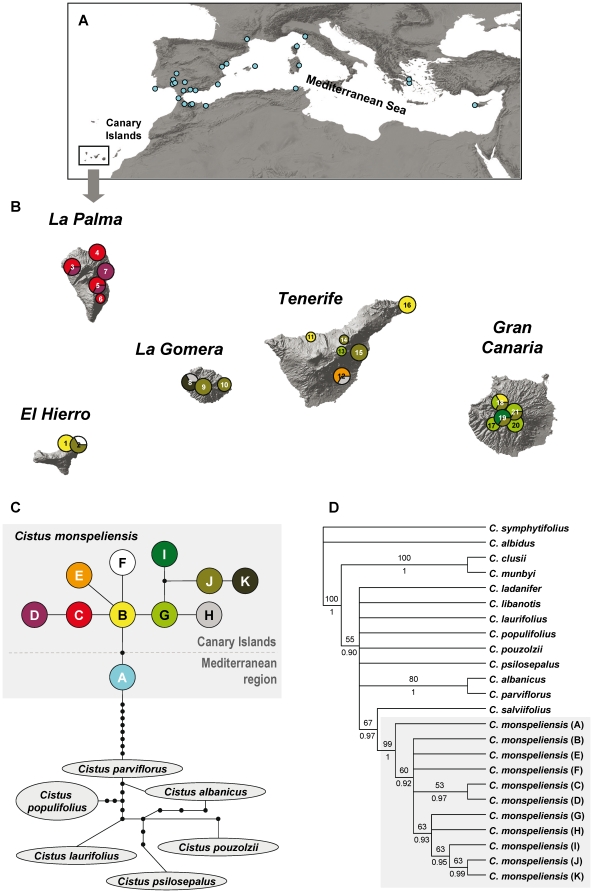
Phylogeographic analysis of *Cistus monspeliensis* based on cpDNA sequences. Sampled populations of *Cistus monspeliensis* in the Mediterranean (A) and the Canary Islands (B) indicating geographical location of the 11 cpDNA haplotypes (colours) inferred from sequences of the *trn*S-*trn*G and *psb*K-*trn*S regions. Chart sizes in B are proportional to the number of sequenced individuals. Canarian populations are numbered as in Table&3146;1. Maps: SRTM Shaded Relief, ESRI. (C) Statistical parsimony network of *C. monspeliensis* haplotypes (indicated by letters) and six closely related species. Lines represent single nucleotide substitutions; dots indicate absent haplotypes (extinct or not found). Colours are as depicted for A and B. (D) Strict consensus tree of the 1322 shortest trees of 89 steps (CI = 0.88; RI = 0.93) from the combined analysis of *trn*S-*trn*G and *psb*K-*trn*S sequences. Numbers above branches are bootstrap values; numbers below branches are Bayesian posterior probabilities.

Among the 17 cpDNA regions tested, two spacers (*trn*S-*trn*G and *psb*K-*trn*S) showed the highest levels of variation. Length of the aligned sequences without the outgroup was 617 bp for *trn*S-*trn*G and 367 bp for *psb*K-*trn*S. The combined analysis of the two regions yielded eleven substitution-based haplotypes of *C. monspeliensis* (Table 2) distributed in the Canary Islands (ten haplotypes, Fig. 1B, Table 1) and the Mediterranean region (one haplotype, Fig. 1A). Haplotype A was found in the 26 Mediterranean populations, as previously reported [30]. Within the Canary Islands, six of the ten haplotypes were exclusive to single islands: C and D to La Palma, E to Tenerife, F to El Hierro, I to Gran Canaria and K to La Gomera. The remaining four haplotypes were shared by two or more islands: B by Tenerife, Gran Canaria and El Hierro, G by Tenerife and Gran Canaria, H by Tenerife and La Gomera, and J by Tenerife, Gran Canaria, La Gomera and El Hierro.

**Table 2:** Variable sites of substitution-based haplotypes found in 47 *C. monspeliensis* populations from the Mediterranean (A) and the Canary Islands (B–K), based on *trn*S-*trn*G (617 bp) and *psb*K-*trn*S (367 bp) sequences.

		DNA region											
		*trn*S*-trn*G sequence position						*psb*K-*trn*S sequence position					
Haplotype	Number of individuals	3	26	357	358	396	412	458	6	14	93	124	365
**A**	26	T	G	G	A	T	A	A	T	A	C	T	A
**B**	8	T	A	T	A	T	A	A	T	A	C	T	A
**C**	8	T	A	T	A	T	A	A	T	A	C	A	A
**D**	5	T	A	T	A	T	A	A	T	T	C	A	A
**E**	2	T	A	T	A	T	A	A	T	A	A	T	A
**F**	1	T	A	T	A	T	A	C	T	A	C	T	A
**G**	9	T	A	T	A	T	A	A	G	A	C	T	A
**H**	2	T	A	T	A	T	C	A	G	A	C	T	A
**I**	2	T	A	T	C	G	A	A	G	A	C	T	A
**J**	14	T	A	T	C	T	A	A	G	A	C	T	G
**K**	2	G	A	T	C	T	A	A	G	A	C	T	G

In the TCS analysis (Fig. 1C) all Canarian haplotypes of *C. monspeliensis* formed a single network with no loops, which was connected to the Mediterranean haplotype (A) through haplotype B. Two mutational steps separated haplotypes A and B. Four clades are connected to haplotype B. The first one is a lineage exclusive to La Palma (C–D). The second and third lineages are tip haplotypes connected to B and only found in Tenerife (E) and El Hierro (F). The fourth lineage is highly differentiated (five haplotypes: G–H–I–J–K) and widely distributed in all islands except for La Palma. Interestingly, unrelated haplotypes are found in the young island of El Hierro (B–F, J) and also in La Gomera (H, J–K).

### Phylogenetic analysis

Combination of *trn*S-*trn*G and *psb*K-*trn*S sequences of 14 *Cistus* species (including those of the eleven haplotypes of *C. monspeliensis*) resulted in an aligned length of 1037 bp. Forty-two of the 73 variable sites from the matrix were phylogenetically informative. MP analysis generated 1322 trees of 89 steps with a consistency index (CI) of 0.88 and a retention index (RI) of 0.93. The strict consensus tree (Fig. 1D) recognizes *C. monspeliensis* populations as monophyletic, with a 99% bootstrap support (BS). The BI phylogeny depicted a congruent topology, with a posterior probability (PP) of 1.00 for the clade of haplotype sequences of the study species. Phylogenetic relationships among haplotypes are congruent with those retrieved in the network analysis. The Mediterranean haplotype (A) is recovered as sister to the Canarian clade, which is supported as monophyletic (60% BS; 0.92 PP).

### Genetic diversity

Tenerife was found to harbour the highest diversity measured in haplotype number (*h* = 5). This island also had the highest haplotypic diversity (*H* = 0.803), followed by El Hierro, Gran Canaria, La Gomera and La Palma (Table 3). Despite showing the lowest diversity, La Palma contained two private haplotypes, while the remaining islands contained one private haplotype each. These results and the high diversity found in the Canary Islands as a whole (*h* = 10; *H* = 0.857) contrast with the lack of diversity in the Mediterranean basin (*h* = 1; *H* = 0.000).

**Table 3:** Genetic diversity parameters across populations of *Cistus monspeliensis* using the *trn*S-*trn*G and *psb*K-*trn*S sequence regions.

	*n*	*h*	*ph*	*H*
**Canary Islands**	53	10	10	0.857
***Tenerife***	12	5	1	0.803
***El Hierro***	6	3	1	0.733
***Gran Canaria***	14	4	1	0.648
***La Gomera***	8	3	1	0.607
***La Palma***	13	2	2	0.513
**Mediterranean region**	26	1	1	0.000

*n*  =  number of sampled individuals; *h*  =  number of substitution-based haplotypes; *ph*  =  number of private haplotypes; *H*  =  haplotypic diversity. Entries are sorted by *H* values.

When comparing haplotype number and haplotypic diversity in monophyletic *Cistus* lineages (Table 4), *C. monspeliensis* populations from the Canary Island yielded the highest values in both estimators. The high cpDNA haplotypic diversity of Canarian *C. monspeliensis* (*H* = 0.857) is only matched by the Canarian purple-flowered lineage of five species (*H* = 0.832). When analysing the *trn*S-*trn*G spacer alone, the highest number of haplotypes (*h* = 6) was found in Canarian populations of *C. monspeliensis* and the Western Mediterranean *C. ladanifer* (Table 4). We found similarly high values of *H* for *trn*S-*trn*G sequences in the Canarian *C. monspeliensis* (H = 0.572), Canarian purple-flowered *Cistus* (H = 0.591) and Mediterranean *C. laurifolius* (H = 0.589).

**Table 4:** Genetic diversity parameters based on cpDNA sequences and Bayesian estimates of stem ages of *Cistus* lineages of the Mediterranean and Canary Islands.

Lineage	cpDNA regions	Stem age (Ma)	*n*	*h*	*H*	Reference
***C. monspeliensis*** ** (Canary Islands)**	*trn*S-*trn*G *psb*K-*trn*S	0.20–0.93	53	10 (6)	0.857 (0.572)	This paper
Purple-flowered *Cistus* (Canary Islands)	*trn*S-*trn*G *trn*K-*mat*K	0.22–1.41	42	7 (4)	0.832 (0.591)	[20]
*C. laurifolius* (Mediterranean)	*trn*S-*trn*G *trn*C-*trn*D *trn*H-*trn*K	0.22–2.18	33	4 (3)	0.646 (0.589)	[30]
Purple-flowered *Cistus* (Mediterranean)	*trn*S-*trn*G *trn*K-*mat*K	0.22–1.41	26	6 (4)	0.640 (0.443)	[20]
*C. ladanifer* (Mediterranean)	*trn*S-*trn*G *trn*K-*mat*K	0.08–1.68	47	8 (6)	0.570 (0.525)	[30], [31]
*C. salviifolius* (Mediterranean)	*trn*S-*trn*G *trn*H-*trn*K	0.08–1.68	52	2 (2)	0.208 (0.208)	[30]
***C. monspeliensis*** ** (Mediterranean)**	*trn*S-*trn*G *psb*K-*trn*S	0.20–0.93	26	1 (1)	0.000 (0.000)	[30, this paper]

*n*  =  number of sampled individuals; *h*  =  number of substitution-based haplotypes; *H*  =  haplotypic diversity. Values of *h* and *H* for the *trn*S-*trn*G spacer are shown in brackets. Entries are sorted by *H* values.

Mediterranean *C. monspeliensis* populations constitute the only analyzed *Cistus* lineage without cpDNA variation of substitution-based haplotypes found to date. A very low variation is also found in *C. salviifolius* (*h* = 2; *H* = 0.208).

### Genetic differentiation

Values of S_nn_ are shown in Table 5. Significant genetic differentiation was retrieved in seven island comparisons: La Palma-Tenerife, La Palma-Gran Canaria, La Palma-El Hierro, La Palma-La Gomera, Tenerife-Gran Canaria, Gran Canaria-El Hierro and Gran Canaria-La Gomera. The highest significant values (S_nn_≈1; p<0.01) were found between La Palma and the other islands.

**Table 5:** Nearest-neighbour statistic (S_nn_) values calculated partitioning the *C. monspeliensis* dataset in order to evaluate genetic differentiation associated with isolation among islands.

	Tenerife	Gran Canaria	La Palma	El Hierro	La Gomera
**Tenerife**	NA	-	-	-	-
**Gran Canaria**	0.658 (p = 0.0150*)	NA	-	-	-
**La Palma**	1.000 (p = 0.0000***)	0.981 (p = 0.0000***)	NA	-	-
**El Hierro**	0.547 (p = 0.3560 ns)	0.738 (p = 0.0120*)	1.000 (p = 0.0010**)	NA	-
**La Gomera**	0.642 (p = 0.0760 ns)	0.760 (p = 0.0060**)	0.971 (p = 0.0000***)	0.690 (p = 0.0520 ns)	NA

ns, not significant;

*, 0.01<p<0.05;

**, 0.001<p<0.01;

***, p<0.001.

### Divergence times and phylogeographic reconstruction

**Figure 2:**
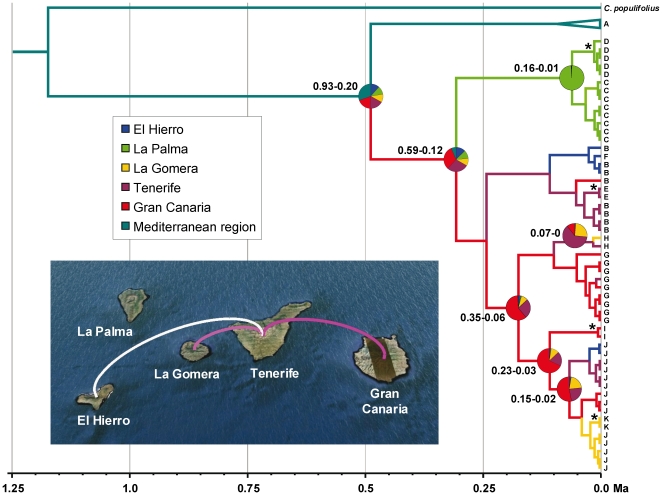
Relaxed molecular-clock chronogram and phylogeographic reconstruction of *Cistus monspeliensis* based on cpDNA sequences. Maximum clade credibility tree produced by analysis of *C. monspeliensis trn*S-*trn*G and *psb*K-*trn*S sequences in BEAST, using *C. populifolius* as the outgroup. Branches are coloured according to the most probable range of their descendent nodes. Pie charts represent posterior probability distributions of ancestral range at well supported (PP>0.95) nodes of interest. 95% highest posterior density intervals for the divergence time estimates of the same nodes are shown. Other nodes with PP>0.95 are indicated with an asterisk (*). Colonization routes supported by a BF>3 are shown on the map. The colour of each route represent its relative support, with darker colours indicating stronger support. The map is based on satellite images available in Google Earth (http://earth.google.com).

According to the parameter analysis in Tracer, number of MCMC iterations of the BEAST analysis was sufficient, with values of effective sample size (ESS) above 400 and plots showing equilibrium after discarding burn-in. The chronogram (Fig. 2) suggests a split between Mediterranean and Canarian populations in the Early to Middle Pleistocene (<930,000 years before present), followed by a stepwise differentiation of Canarian lineages in the last c. 600,000 years. No further haplotype differentiation was detectable in the Mediterranean lineage for the same period. The discrete phylogeographic analysis (Fig. 2) yielded a high uncertainty on the range of the common ancestor of all *C. monspeliensis* sequences, although the Mediterranean region received the highest probability (0.31). Two islands were similarly supported as the most likely ancestral range of all Canarian samples: Gran Canaria (0.32) and Tenerife (0.29). The ancestor of the lineage formed by haplotypes G-K probably inhabited Gran Canaria (0.61) no longer than 350,000 years ago. Despite uncertainty on topology and direction of colonization events, the MCC tree supports a single colonization of La Palma, two colonizations of El Hierro, two colonizations of La Gomera and several exchanges between Gran Canaria and Tenerife. Three migration routes were supported by the BF test (Fig. 2): Gran Canaria-Tenerife (BF = 23.20), Tenerife-La Gomera (BF = 9.68) and Tenerife-El Hierro (BF = 3.74).

## Discussion

Artificial crossings of four species (*C. parviflorus*, *C. laurifolius*, *C. libanotis*, *C. ladanifer*) of the white-flowered lineage revealed maternal inheritance of plastid haplotypes [31]. Thus, phylogeographic reconstruction of plastid haplotypes reflects plant colonization by seeds. Despite the absence of a special dispersal mechanism, successful colonization by *Cistus* is supported by species distribution and phylogeographic reconstructions [20], [30], [31].

### The continent-island connection

The geographical distribution of Canarian-mainland species can theoretically be attributed to human-mediated introduction, natural colonization from the continent or natural colonization from the Canary Islands. We can rule out human introduction based on herein findings of ten haplotypes endemic to the Canary Islands. This result is consistent with some degree of morphological differentiation found in Gran Canaria (*Cistus grancanariae* [29]). Our Bayesian phylogeographic reconstruction is not conclusive regarding the ancestral area of *C. monspeliensis*, although the most likely range is the Mediterranean region. Indeed, the network analysis suggests colonization of the Canary Islands followed by differentiation into ten haplotypes from the ancestral haplotype exclusively distributed across the Mediterranean (Fig. 1C, D).

Lower genetic variation is expected in islands due to founder effects [9], [18], [51]. In addition, larger population size and wider geographical range of *C. monspeliensis* in the Mediterranean basin would lead us to expect a higher genetic variation in mainland under similar historical processes. However, our results do not fit these expectations. To our knowledge, *C. monspeliensis* is the first example of a plant with much higher genetic variation in populations of an oceanic archipelago as compared to the mainland (reviewed in Table 6). Finding of similar or higher levels of genetic variation on islands as compared to continents has been variably ascribed to: (1) multiple continent-island colonizations; (2) species formation on the islands and subsequent colonization to the continent; and (3) genetic bottlenecks in the continent [13], [16], [17], [52]. For *C. monspeliensis*, our haplotype network and the monophyly of Canarian haplotypes in the phylogenetic reconstruction support a single colonization of the Canary Islands from the continent (Fig. 1). The island-to-continent colonization hypothesis [22] is not supported given the derived condition of all Canarian haplotypes. Therefore, genetic bottleneck in the continent is the most plausible hypothesis to explain our results. Indeed, white-flowered species of *Cistus* display various haplotypes across the Mediterranean basin, except for *C. monspeliensis* [30], [[31], Table 4].

**Table 6:** Population genetic and phylogeographic studies in which mainland and oceanic island populations of the same plant species (or very closely related species) were examined.

Taxon	Oceanic island(s)	Mainland	Markers	Pattern	Reference
*Asplenium hookerianum*	Chatham Islands	New Zealand	cpDNA haplotypes	Multiple colonizations (but still lower variation in oceanic islands).	[15]
*Campanula punctata*	Izu Islands	Honsu	Allozymes	Lower variation in oceanic islands.	[10]
*Cistus monspeliensis*	Canary Islands	Mediterranean region	cpDNA haplotypes	Much higher variation in oceanic islands.	This paper
*Clidemia hirta* *	Hawaii	Costa Rica	Allozymes	Low variation both in oceanic islands and mainland.	[64]
*Hibiscus tiliaceus*	Bonin Islands, Hawaii, Mariana, Samoa	SW Asia	cpDNA haplotypes	Lower variation in oceanic islands.	[65]
*Homalothecium sericeum*	Madeira	Europe, North Africa	nrDNA haplotypes	Low haplotypic variation in oceanic islands.	[66]
*Laurus nobilis* complex	Canary Islands, Madeira, Azores	Mediterranean region	cpDNA haplotypes	Lower variation in oceanic islands: one single haplotype shared with North Africa.	[24]
*Limonium wrightii*	Daito Islands	Ryukyu Islands	nrDNA haplotypes	Lower variation in oceanic islands.	[67]
*Olea europaea*	Canary Islands, Madeira	Mediterranean region, northern Africa	AFLP, cpDNA haplotypes	Lower variation in oceanic islands.	[14], [25]
*Pterocarpus officinalis*	Guadeloupe	North and Central America, Caribbean	AFLP	Low variation in oceanic island.	[12], [68]
*Rubus alceifolius* *	Mayotte, La Réunion, Mauritius	SE Asia, Madagascar	AFLP	Multiple introductions in Madagascar, but low variation in oceanic islands.	[17]

Continental islands are regarded as mainland. Mediterranean-Macaronesian examples are underlined.

*Human-mediated introduction in oceanic islands.

A general pattern of the evolutionary history of *Cistus* in oceanic islands is difficult to be described. In the purple-flowered lineage, similar haplotype diversity has been originated in the Mediterranean (three species) and Canarian (five species) sublineages since the Early to Middle Pleistocene [20]. In contrast, a single white-flowered species (*C. monspeliensis*) displays higher levels of haplotype differentiation (ten haplotypes) than those of the five purple-flowered species (seven haplotypes) in the Canary Islands. Our phylogenetic analysis and estimates of divergence times of *C. monspeliensis* suggest that this haplotype diversity was generated in the archipelago in the last 600,000 years, but little morphological differentiation has taken place (Fig. 2).

Climate changes in the Pleistocene may account for a massive extinction of *C. monspeliensis* haplotypes in the Mediterranean, which was followed by postglacial colonization by a single, ancient haplotype across the basin [30]. This pattern of extinction and recolonization has previously been described for *Pinus pinea* [53]. Although our result should be confirmed using additional DNA markers (specifically nuclear markers), the clear difference in cpDNA haplotype variation of *C. monspeliensis* is consistent with the canonical hypothesis of impoverishment or extinction of populations in the continent in the last glacial period and survival in the Canarian archipelago due to climatic buffering in Atlantic islands [54], [55]. However, a pattern supporting this hypothesis has seldom been documented using phylogenetic and phylogeographic approaches, in contrast to paleobotanical evidence. Remarkable plant survival has been interpreted in woody taxa such as *Pinus canariensis*, *Ocotea*, *Persea* and *Dracaena*, which are inhabitants of the Canary Islands, but became extinct in the continent between the end of the Tertiary and the Quaternary periods, as documented by European macrofossils [56], [57].

### Active colonization across the Canary Islands

As already reported in the Mediterranean Basin [30], [31], *Cistus* displayed an unexpected capacity for long distance dispersal across the Canary Islands. A temporal “stepping-stone” pattern of colonization, since new island formation, has been described in oceanic archipelagos [58], [59]. This pattern was not found in *C. monspeliensis*, as the original colonization of the Canary Islands (<930,000 years) may have postdated the formation of the youngest island (El Hierro: 1.12 Ma [60]). Alternatively, a geographical stepping-stone pattern of colonization from east to west could be plausible, based on the arrangement of this archipelago in relation to the mainland [59], [61]. In our case, Tenerife and Gran Canaria contain the highest numbers of *C. monspeliensis* haplotypes (five and four respectively), including the ancestral one, and received the highest probabilities as the ancestral range of Canarian lineages (Fig. 2). These are the closest islands to the continent with *Cistus* populations and contain the largest areas of thermophilous habitats suitable for *C. monspeliensis*. We hypothesize that Tenerife and Gran Canaria may have constituted the center of diversification of current Canarian lineages. Colonization of La Gomera and El Hierro occurred via long-distance dispersal from Tenerife to the southwest, as supported by our phylogeographic analysis. The early colonization of La Palma may have occurred from Tenerife or Gran Canaria, but the former seems more likely given its geographical closeness. This colonization pattern is similar to that described for the endemic *Pinus canariensis* [62]. The easy of *C. monspeliensis* dispersal is observed in our haplotype network, phylogeographic reconstruction and S_nn_ values, which are consistent with a double colonization of La Gomera and El Hierro. Multiple colonization events between Tenerife and Gran Canaria are also inferred, although the directionality of the dispersal cannot be clearly determined based on our analyses. In contrast, it appears that there was a single colonization of La Palma, which exclusively harbours two connected haplotypes. Volcanic activity in the Canary Islands may have favoured extinction of certain populations of *C. monspeliensis*, followed by isolation, differentiation and recolonization from different source areas. In fact, the ancestral haplotype has been detected in the populations of Anaga (northeast Tenerife) and nearby Teno massif (northwest Tenerife), which are old areas considered to have been independent islands for millions of years [14], [63], while derived tip haplotypes are found in younger areas of Tenerife.

In summary, the dispersal capacity of *Cistus*, initially suggested by the presence of two independent lineages in the Canary Islands [19], is further supported by evidence of the native status and active colonization of *C. monspeliensis* across the archipelago. The phylogeographic relationships not only within the purple- but also within the white-flowered lineage indicate that the current distribution of Canarian *Cistus* populations reflects recurrent dispersal events [20, this paper]. It remains to be determined why significant speciation occurred in the purple-flowered lineage (five endemic species), whereas it was more limited in the white-flowered lineage (see *C. grancanariae* [29]) during a similar period of time (<900,000 years [20, this paper]).

## Supporting Information

Table S1 *Cistus* species and populations used for sequencing plastid regions (*trn*S-*trn*G and *psb*K-*trn*S) and GenBank accession numbers. Taxonomy follows that of [19], except for *C. albanicus* (formely called *C. sintenisii*).
(DOC)
