# Supplementary material for: Genetically Depauperate in the Continent but Rich in Oceanic Islands: Cistus monspeliensis (Cistaceae) in the Canary Islands
Source: PLoS One. 2011 Feb 14;6(2):e17172. doi: 10.1371/journal.pone.0017172 (PMC3038934; doi:10.1371/journal.pone.0017172)
Supplement: Table S1 — Cistus species and populations used for sequencing plastid regions (trnS-trnG and psbK-trnS) and GenBank accession numbers. Taxonomy follows that of [19], except for C. albanicus (formely called C. sintenisii). (DOC) [file pone.0017172.s001.doc]

| **Taxon** | **(Population number) Locality** | **Voucher** | **Haplotype code** | ***trn*S-*trn*G accession no.** | **psbK-trnS accession no.** |
| --- | --- | --- | --- | --- | --- |
| *Cistus* L. |  |  |  |  |  |
| *Cistus albanicus* E.F. Warb. ex Heywood | Cultivated | R. G. Page 8cBGA04 (MA) | - | FJ228736 | GU289172 |
| *Cistus albidus* L. | Morocco, Tetuán | P. Vargas 41PV03 (MA) | - | GQ927042 | - |
| “ | Portugal, Sagres | B. Guzmán 34BGA04 (MA) | - | - | GU289173 |
| *Cistus clusii* Dunal | Spain, Málaga, Mijas | R. G. Page 8bBGA04 (MA) | - | FJ228739 | GU289174 |
| *Cistus ladanifer* L. | Spain, Madrid, Boadilla | B. Guzmán 7BGA03 | - | FJ189421 | GU289175 |
| *Cistus laurifolius* L. | Morocco, Bab-Berred | V. Valcárcel 21VV03 (MA) | - | GU288879 | GU289140 |
| *Cistus libanotis* L. | Spain, Córdoba | B. Guzmán 35BGA04 (MA) | - | FJ228732 | - |
| “ | Cultivated | O. Filippi 5BGA04 (MA) | - | - | GU289176 |
| *Cistus monspeliensis* L. | (1) Spain, Canary Islands, El Hierro, La Peña | C. García-Verdugo 39CG05 (MA) | B/B/B | HQ286791 HQ286792 HQ286793 | HQ286844 HQ286845 HQ286846 |
| “ | (2) Spain, Canary Islands, El Hierro, Valverde | C. García-Verdugo 41CG05 (MA) | F/J/J | HQ286794 HQ286795 HQ286796 | HQ286847 HQ286848 HQ286849 |
| “ | (3) Spain, Canary Islands, La Palma, Barranco Garome | C. García-Verdugo 34CG05 (MA) | C/C/D | HQ286797 HQ286798 HQ286799 | HQ286850 HQ286851 HQ286852 |
| “ | (4) Spain, Canary Islands, La Palma, La Tosca | B. Guzmán 142BGA04 (MA) | C/C/C | HQ286800 HQ286801 HQ286802 | HQ286853 HQ286854 HQ286855 |
| “ | (5) Spain, Canary Islands, La Palma, Los Llanos – Santa Cruz | V. Valcárcel 63VV04 (MA) | C/C/D | HQ286803 HQ286804 HQ286805 | HQ286856 HQ286857 HQ286858 |
| “ | (6) Spain, Canary Islands, La Palma, Villa de Mazo | P. Vargas 254PV02 (MA) | C | HQ286806 | HQ286859 |
| “ | (7) Spain, Canary Islands, La Palma, Barranco Seco | C. García-Verdugo 26CG05 (MA) | D/D/D | HQ286807 HQ286808 HQ286809 | HQ286860 HQ286861 HQ286862 |
| “ | (8) Spain, Canary Islands, La Gomera, Arure | C. García-Verdugo 19CG05 (MA) | H/K/K | HQ286810 HQ286811 HQ286812 | HQ286863 HQ286864 HQ286865 |
| “ | (9) Spain, Canary Islands, La Gomera, Alto de Garajonay | A. Herrero AH2443 (MA) | J/J/J | HQ286813 HQ286814 HQ286815 | HQ286866 HQ286867 HQ286868 |
| “ | (10) Spain, Canary Islands, La Gomera, Jaragán | C. García-Verdugo 10CG05 (MA) | J/J | HQ286816 HQ286817 | HQ286869 HQ286870 |
| “ | (11) Spain, Canary Islands, Tenerife, Barranco de las Ánimas | B. Guzmán 12BGA05 (MA) | B | HQ286818 | HQ286871 |
| “ | (12) Spain, Canary Islands, Tenerife, Villa de Arico | P. Vargas 42PV05 (MA) | E/E/H | HQ286819 HQ286820 HQ286821 | HQ286872 HQ286873 HQ286874 |
| “ | (13) Spain, Canary Islands, Tenerife, road to Teide | B. Guzmán 14BGA05 (MA) | G | HQ286822 | HQ286875 |
| “ | (14) Spain, Canary Islands, Tenerife, Aguamansa | C. García-Verdugo 8CG05 (MA) | J | HQ286823 | HQ286876 |
| “ | (15) Spain, Canary Islands, Tenerife, Güímar | C. García-Verdugo 54PV05 (MA) | J/J/J | HQ286824 HQ286825 HQ286826 | HQ286877 HQ286878 HQ286879 |
| “ | (16) Spain, Canary Islands, Tenerife, Igüeste de San Andrés | P. Vargas 56PV05 (MA) | B/B/B | HQ286827 HQ286828 HQ286829 | HQ286880 HQ286881 HQ286882 |
| “ | (17) Spain, Canary Islands, Gran Canaria, Embalse del Mulato | B. Guzmán 1BGA05 (MA) | G/G | HQ286830 HQ286831 | HQ286883 HQ286884 |
| “ | (18) Spain, Canary Islands, Gran Canaria, Artenara | P. Vargas 61PV05 (MA) | B/G/J | HQ286832 HQ286833 HQ286834 | HQ286885 HQ286886 HQ286887 |
| “ | (19) Spain, Canary Islands, Gran Canaria, road GC-605 | B. Guzmán 3BGA05 (MA) | I/I/J | HQ286835 HQ286836 HQ286837 | HQ286888 HQ286889 HQ286890 |
| “ | (20) Spain, Canary Islands, Gran Canaria, San Bartolomé de Tirajana | B. Guzmán 6BGA05 (MA) | G/G/G | HQ286838 HQ286839 HQ286840 | HQ286891 HQ286892 HQ286893 |
| “ | (21) Spain, Canary Islands, Gran Canaria, Roque Nublo | P. Vargas 169PV08 (MA) | G/G/J | HQ286841 HQ286842 HQ286843 | HQ286894 HQ286895 HQ286896 |
| “ | Morocco, Tánger | P. Vargas 30PV03 (MA) | A | GU288910 | GU289144 |
| “ | Morocco, Xauen | P. Vargas 206PV06 (MA) | A | GU288911 | GU289145 |
| “ | Morocco, Targuist | B. Guzmán 110BGA04 (MA) | A | GU288912 | GU289146 |
| “ | Morocco, Tafouralt | B. Guzmán 88BGA04 (MA) | A | GU288913 | GU289147 |
| “ | Morocco, Beni Hadifa | B. Guzmán 100BGA04 (MA) | A | GU288914 | GU289148 |
| “ | Tunisia, Sejenane | J. J. Aldasoro 2802A (MA) | A | GU288915 | GU289149 |
| “ | Portugal, Sagres | B. Guzmán 35BGA04 (MA) | A | GU288916 | GU289150 |
| “ | Spain, Huelva, Zalamea la Real | P. Vargas 224PV06 (MA) | A | GU288917 | GU289151 |
| “ | Spain, Badajoz, Fregenal de la Sierra | P. Vargas 218PV06 (MA) | A | GU288918 | GU289152 |
| “ | Spain, Huelva, Zufre | B. Guzmán 12BGA04 (MA) | A | GU288919 | GU289153 |
| “ | Spain, Badajoz, Cornalvo reservoir | P. Vargas 218PV06 (MA) | A | GU288920 | GU289154 |
| “ | Spain, Cádiz, Sierra de Grazalema | P. Vargas 255PV06 (MA) | A | GU288921 | GU289155 |
| “ | Spain, Córdoba, Posadas – Villaviciosa | B. Guzmán 57BGA04 (MA) | A | GU288922 | GU289156 |
| “ | Spain, Málaga, Casabermeja | P. Vargas 18PV06 (MA) | A | GU288923 | GU289157 |
| “ | Spain, Granada, Almuñécar | P. Vargas 14PV06 (MA) | A | GU288924 | GU289158 |
| “ | Spain, Murcia, Portman | M. Fernández-Mazuecos & D. Orgaz 2MF08 (MA) | A | GU288925 | GU289159 |
| “ | Spain, Castellón, Chóvar | B. Guzmán 42BGA03 (MA) | A | GU288926 | GU289160 |
| “ | Spain, Castellón, Alcocebre | P. Vargas 117PV07 (MA) | A | GU288927 | GU289161 |
| “ | Balearic Islands, Menorca, Punta Galdana | P. Vargas 324PV02 (MA) | A | GU288928 | GU289162 |
| “ | France, Saint-Chinian | P. Vargas 231PV06 (MA) | A | GU288929 | GU289163 |
| “ | Corsica, Barchetta | M. Escudero & M. Luceño 73ME07 (MA) | A | GU288930 | GU289164 |
| “ | Italy, San Giuliano Terme | M. Fernández-Mazuecos 30MF07 (MA) | A | GU288931 | GU289165 |
| “ | Sardinia, Baunei | A. Quintanar MA709533 | A | GU288932 | GU289166 |
| “ | Greece, Saronida | P. Vargas 66PV08 (MA) | A | GU288933 | GU289167 |
| “ | Greece, Kapandriti | P. Vargas 75PV08 (MA) | A | GU288934 | GU289168 |
| “ | Cyprus, Peyia | Iter Mediterraneum IV MA495340 | A | GU288935 | GU289169 |
| *Cistus munbyi* Pomel | Morocco | R. G. Page 8BGA04 (MA) | - | FJ228738 | GU289177 |
| *Cistus parviflorus* Lam. | Crete | R. G. Page 151BGA04 (MA) | - | GQ281683 | GU289178 |
| *Cistus populifolius* L. | Spain, Ávila, Arenas de San Pedro | R. G. Page 8tBGA04 (MA) | - | - | GU289179 |
| “ | Portugal, Ourique | P. Vargas 5PV03 (MA) | - | GQ281692 | - |
| *Cistus pouzolzii* Delile | France | R. G. Page 8tBGA04 (MA) | - | FJ228734 | GU289180 |
| *Cistus psilosepalus* Sweet | Spain, Ávila, Arenas de San Pedro | P. Vargas 6PV03 (MA) | - | FJ228737 | GU289181 |
| *Cistus salviifolius* L. | Crete, Vlatos – Elos | P. Vargas 117PV05 (MA) | - | GU288984 | GU289171 |
| *Cistus symphytifolius* Lam. | Spain, Canary Islands, Tenerife, Vilaflor | P. Vargas 174PV05 (MA) | - | GQ927083 | GU289182 |
